# Evaluating the Usefulness of Translation Technologies for Emergency Response Communication: A Scenario-Based Study

**DOI:** 10.2196/11171

**Published:** 2019-01-28

**Authors:** Anne M Turner, Yong K Choi, Kristin Dew, Ming-Tse Tsai, Alyssa L Bosold, Shuyang Wu, Donahue Smith, Hendrika Meischke

**Affiliations:** 1 School of Public Health Department of Health Services University of Washington Seattle, WA United States; 2 School of Medicine Department of Biomedical Informatics and Medical Education University of Washington Seattle, WA United States; 3 College of Engineering Department of Human Centered Design and Engineering University of Washington Seattle, WA United States

**Keywords:** Chinese, Emergency Medical Services, emergency response, language barriers, language translation, public health informatics, Spanish, limited English proficient, translation technologies

## Abstract

**Background:**

In the United States, language barriers pose challenges to communication in emergency response and impact emergency care delivery and quality for individuals who are limited English proficient (LEP). There is a growing interest among Emergency Medical Services (EMS) personnel in using automated translation tools to improve communications with LEP individuals in the field. However, little is known about whether automated translation software can be used successfully in EMS settings to improve communication with LEP individuals.

**Objective:**

The objective of this work is to use scenario-based methods with EMS providers and nonnative English-speaking users who identified themselves as LEP (henceforth referred to as LEP participants) to evaluate the potential of two automated translation technologies in improving emergency communication.

**Methods:**

We developed mock emergency scenarios and enacted them in simulation sessions with EMS personnel and Spanish-speaking and Chinese-speaking (Mandarin) LEP participants using two automated language translation tools: an EMS domain-specific fixed-sentence translation tool (QuickSpeak) and a statistical machine translation tool (Google Translate). At the end of the sessions, we gathered feedback from both groups through a postsession questionnaire. EMS participants also completed the System Usability Scale (SUS).

**Results:**

We conducted a total of 5 group sessions (3 Chinese and 2 Spanish) with 12 Chinese-speaking LEP participants, 14 Spanish-speaking LEP participants, and 17 EMS personnel. Overall, communications between EMS and LEP participants remained limited, even with the use of the two translation tools. QuickSpeak had higher mean SUS scores than Google Translate (65.3 vs 48.4; *P*=.04). Although both tools were deemed less than satisfactory, LEP participants showed preference toward the domain-specific system with fixed questions (QuickSpeak) over the free-text translation tool (Google Translate) in terms of understanding the EMS personnel’s questions (Chinese 11/12, 92% vs 3/12, 25%; Spanish 12/14, 86% vs 4/14, 29%). While both EMS and LEP participants appreciated the flexibility of the free-text tool, multiple translation errors and difficulty responding to questions limited its usefulness.

**Conclusions:**

Technologies are emerging that have the potential to assist with language translation in emergency response; however, improvements in accuracy and usability are needed before these technologies can be used safely in the field.

## Introduction

The United States is linguistically diverse, with over 350 spoken languages [1]. In 2016, approximately 63.2 million US residents spoke a language other than English [2], and approximately 40% of these individuals (25.4 million people) are considered limited English proficient (LEP) [3]. LEP is defined as having a primary language that is not English and limited ability to read, speak, write, or understand English [4].

With growth of the foreign-born population in the United States, the number of LEP individuals is also growing [5]. From 1990 to 2010, the number of LEP individuals in the United States increased by 80%, meaning that in 2010, about 25.2 million or 9% of the US population over the age of 5 years was considered LEP [5].

Health care providers in many parts of the country likely experience challenges with language translation on a frequent basis. In hospital settings, language barriers have contributed to disparities in care for LEP individuals, including longer hospital stays, greater risk of hospital-acquired infections, and increased likelihood of readmission after discharge [6]. In worst-case scenarios, LEP individuals are misdiagnosed and experience serious consequences from improper or delayed treatment [7]. In the emergency response setting, lack of clear communication between LEP individuals and Emergency Medical Services (EMS) personnel can interfere with prompt and accurate dispatching of aid [8]. Language barriers were listed as the second most common reason for delay in care delivery among EMS providers in Minnesota [9]. While the use of interpreters and telephone language lines are recommended in emergency situations involving LEP individuals, time constraints and perceived delays in connecting with interpreters present barriers to their use.

With the advent of new technologies, options for communication with LEP individuals are expanding. A variety of automated translation tools have been developed to assist with translation and interpretation between individuals that have language incongruence. In one study, EMS personnel report using digital applications on their personal mobile devices, such as Google Translate (a freely available Web-based system developed for general translation use), to attempt to communicate with their patients [10]. Many fire departments are also using electronic tools in the field, such as tablets [11]. Access to tablets in the field has opened the door to the use of other translation software. For example, some EMS departments are considering the use of “QuickSpeak,” a tablet-based translation app and one of the few translation tools designed specifically for use in emergency response.

Although digital communication devices could be promising, these tools have not been systematically evaluated for use in the field by EMS, and there is little to no evidence regarding the usefulness of these newer strategies in facilitating communication between LEP individuals and EMS providers. Our prior work and review of the literature has revealed that in clinical or public health settings, most automated translation systems are not accurate enough to be safely used [12-19].

This study takes place in King County, Washington, where in a recent survey, 78% (96/123) of 911 dispatchers reported that communication difficulties with LEP individuals affect the medical care these callers receive [20]. In Washington, Spanish and Chinese (Mandarin) are the two most commonly spoken non-English languages. Among non-English speakers in the United States, Chinese and Spanish-speaking individuals are also some of the most likely to have limited English proficiency [21]. Many EMS agencies in the King County area are considering the use of automated language tools to improve communication with LEP individuals but are concerned about the safety of these tools in the field. The purpose of this study was to gather evidence on how QuickSpeak and Google Translate (which were both being considered for use by EMS personnel in King County) performed in emergency situations where clear communication is critical for rapid identification, treatment, and transport of patients. Specifically, we tested how QuickSpeak and Google Translate performed in mock emergency response settings requiring prompt EMS response and translation from English to Spanish and Chinese (Mandarin).

## Methods

### Participants

For our study, we focused on the two most common languages spoken by LEP individuals in the King County area: Spanish and Chinese (Mandarin). We recruited Spanish- and Chinese-speaking individuals whose native language was not English and who self-identified as LEP (henceforth referred to as LEP participants) from local community organizations in King County, Washington. Bilingual research team members collaborated with community organization staff from programs serving LEP individuals in King County to recruit participants. Additionally, we used convenience sampling, through research team members’ personal contacts, to enhance recruitment. To be eligible for the study, LEP participants had to be 18 years or older, speak at least some English but identify themselves as having difficulty communicating in English, and prefer to receive medical care in their native language (Spanish or Chinese) [22]. Given the challenges of recruiting and collaborating with LEP individuals, we sought to minimize the burden of participant screening procedures and did not add a quantitative language assessment instrument to the screening. The University of Washington Institutional Review Board approved all study protocols and materials.

We recruited EMS personnel, including on-duty fire fighters and emergency medical technicians, from local fire departments in King County through convenience sampling. The research team contacted battalion chiefs at local fire departments located in close proximity to communities where there are a large number of LEP residents and asked for permission to recruit firefighters and conduct a simulation session at their station. We based the number of sessions on when data saturation was reached and additional responses were not forthcoming [23].

### Tools

The King County Vulnerable Populations Strategic Initiative [24] team identified two translation tools, QuickSpeak and Google Translate, which EMS personnel were piloting for use (QuickSpeak) or were using on rare occasions (Google Translate). We investigated the potential use of these tools for improving communication between EMS and LEP individuals through simulation sessions involving emergency scenarios.

#### QuickSpeak

**Figure 1:**
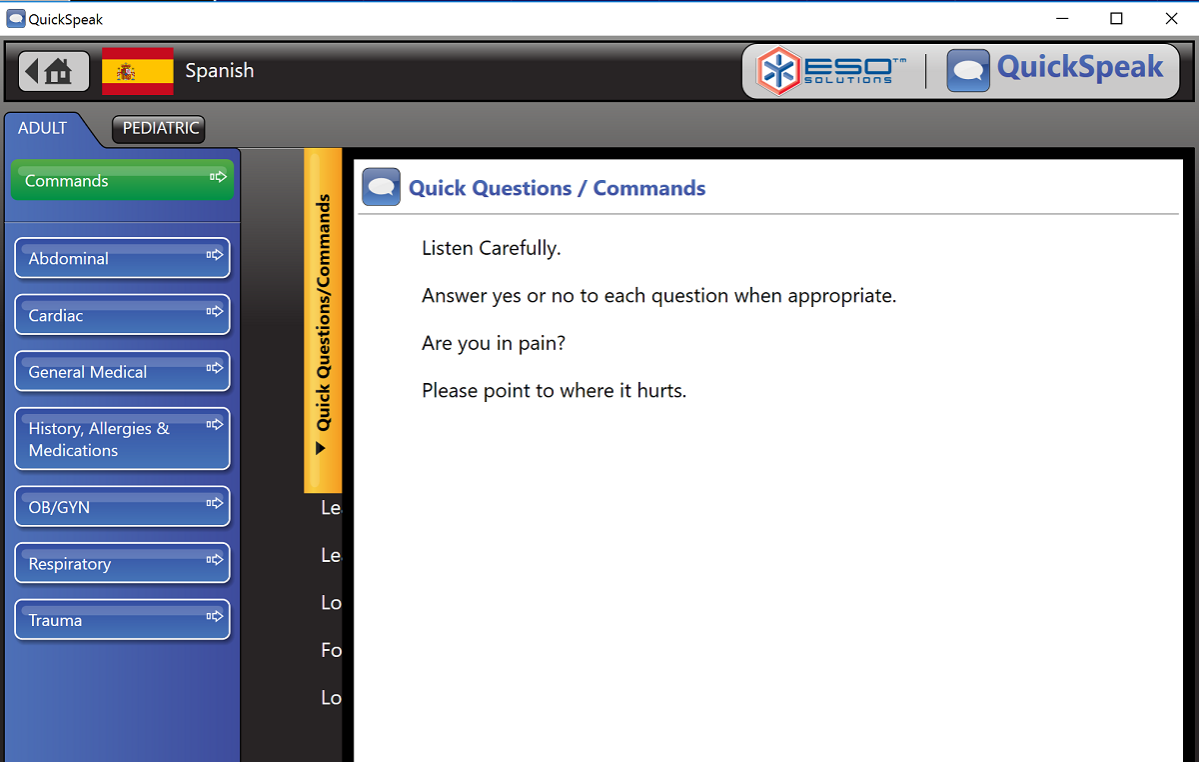
Screenshot of QuickSpeak translation software. (Source: www.esosolutions.com).

**Figure 2:**
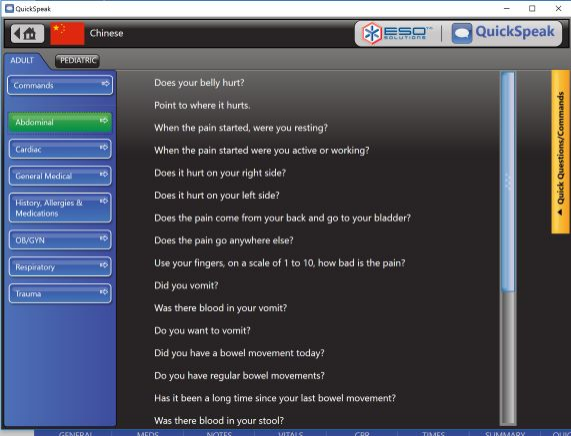
Screenshot of QuickSpeak translation software (Source: www.esosolutions.com).

QuickSpeak is an EMS domain-specific translation software that provides EMS personnel access to internally validated, verbal translations of a set of standard English questions asked by first responders. QuickSpeak is one of the few translation tools designed specifically for emergency response. The EMS personnel can select written questions using a touchscreen, and the software provides recorded translations in the requested language. All questions are posed in a yes or no response format. Questions and answers are not recorded or archived. At the time of this study, QuickSpeak could respond in 7 languages: Spanish, Italian, French, German, Finnish, Chinese (Mandarin), and Vietnamese. Figures 1 and 2 present screenshots of QuickSpeak.

#### Google Translate

**Figure 3:**
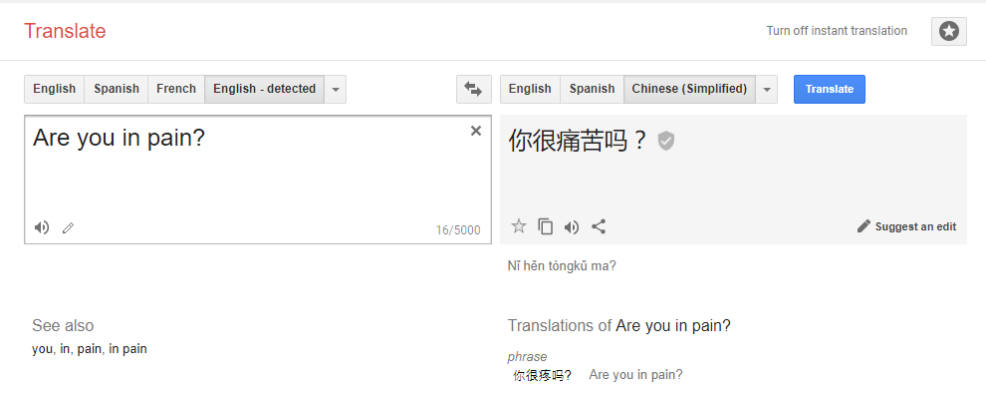
Screenshot of Google Translate software (Source: translate.google.com).

Google Translate is a free, Web-based and app-based translation software that allows users to write free text in one language and have it converted to written or spoken text in another language. Google Translate utilizes a statistics-based translation (statistical machine translation) method that produces translations based on their probability of being correct [25]. Currently, Google Translate can translate over 100 languages. Google Translate has been used in many machine translation studies for comparison [26,27]. Figure 3 presents a screenshot of Google Translate.

### Study Design

Because field evaluation of new translation tools poses logistical and ethical issues, we drew from scenario-based design to guide this research. Scenario-based design is a key approach to testing and comparing the usefulness of new technologies under “controlled” but realistic conditions [28,29]. In scenario-based design, potential technology users assess the value of technology through participation as actors in realistic, scripted situations. The scripted situations, or scenarios, are developed based on knowledge of actual events, revealed through interviews, focus groups, or observations with the potential technology users.

For our study, 2 research team members (AT, a pediatrician and MT, an emergency medicine physician) created three pairs of scenarios, based on their experience and prior review of transcripts from real-life emergency calls involving LEP.

Scenarios were developed to illustrate a common situation occurring during an EMS response and described the information EMS responders need from LEP individuals. Each scenario described the emergency situation, the “patient” and “support person” (family or friend), precipitating events, the “patient’s” medications and allergies, and basic “patient” demographics, such as age and occupation. We created a pair of similar but not identical scenarios to test and compare the two translation tools.

A battalion chief from a local fire department reviewed the scenarios to ensure that they reflected realistic situations. In response to the review, we made minor modifications. Bilingual research team members translated completed scenarios into traditional Chinese and Spanish. Multimedia Appendix 1 presents the scenario pairs created for this study.

To test the feasibility and usability of QuickSpeak and Google Translate, we held group simulation sessions with Spanish-speaking and Chinese-speaking (Mandarin) LEP participants and EMS personnel at locations convenient for the participants. There were 2 language-appropriate bilingual facilitators who recruited participants and organized the sessions. Table 1 provides a summary of the sessions.

At the beginning of each session, the bilingual facilitator obtained informed consent from LEP participants; collected demographic data including age, education level, number of years in the United States, and self-identified spoken and written English proficiency levels; and explained the overall goals of the evaluation and the language technologies.

Prior to the session, we appraised the LEP participants of the scenario and their role. Working in pairs, one LEP participant played the role of a “patient,” responding to the EMS provider’s questions with the assistance of either QuickSpeak or Google Translate; the other LEP participant served in the role of a friend or relative “support person.” We provided the EMS participants with information similar to what they would receive from a 911 dispatcher, such as the “patient’s” address, age, chief complaint, and primary language. We did not give the EMS personnel information regarding the underlying health issue that the LEP ‘‘patient’’ was acting out.

Each LEP participant acted in the role of a “patient” or “support person” in scenarios involving each of the two technologies. The EMS personnel sought answers to key questions, such as the chief complaint, symptoms, and medications.

### Measures

At the end of the session, EMS personnel and LEP participants filled out postsession questionnaires, providing feedback on the translation technologies (see Multimedia Appendix 2). The EMS personnel questionnaire included qualitative feedback questions to gather their impressions and experiences with the translation technologies in their own words. For example, it asked them to compare the two technologies (Google Translate and QuickSpeak), identify problems they experienced, and suggest changes. It also collected information on their prior experiences using translation technologies during an emergency.

LEP questionnaires were translated into Spanish and Chinese by native-speaking bilingual research members. The LEP questionnaire asked similar qualitative questions about the participants’ experiences using the translation technology, the problems they encountered, and whether they had ever needed translation during a medical emergency.

The EMS participants were also asked to evaluate the usability of the technologies using a System Usability Scale (SUS) instrument [30]. The SUS generates a quantitative measure of usability through 10 5-point, Likert-type questions, where participants provide their level of agreement or disagreement. The SUS is employed widely for assessing the perceived usability of technologies including mobile apps and monitoring devices for health care [30-33], and it has demonstrated validity, reliability, and sensitivity in numerous studies [34-36]. Since the SUS measures usability, it was only administered to EMS participants, as they were the primary user group operating the translation tools, and LEP participants were not handling the translation technologies and driving the interactions.

LEP participants received a US $25 honorarium for participation in our study, but as paid professionals, EMS participants could not accept honorariums.

**Table 1:** Overview of simulation sessions.

Sessions	Location	Limited English proficient participants, n (%)	Emergency Medical Services personnel, n (%)
Spanish #1	Local fire department	6 (23)	6 (35)
Spanish #2	Local fire department	8 (31)	6 (35)
Chinese #1	Research office	4 (15)	1 (6)
Chinese #2	Chinese group home residence	4 (15)	1 (6)
Chinese #3	Local fire department	4 (15)	4 (25)

### Data Analysis

We used thematic analysis to examine qualitative responses to open-ended questions on the postsession questionnaires. The practical research question of whether automated language translation tools can facilitate LEP communication in emergency settings drove our thematic analysis. Researchers (YKC, KD, SW, DS) coded the questionnaire responses independently and then met to discuss identified codes and themes. Through several rounds of discussion, we reconciled differences and grouped similar codes to formulate meaningful thematic categories [37].

We conducted descriptive statistical analysis of quantitative data using R software (R Foundation for Statistical Computing) [38]. We also used a Mann-Whitney U test to investigate the relationship between SUS scores and the two technology tools evaluated.

## Results

### Participants

We held 5 group simulation sessions (3 Chinese and 2 Spanish) with 12 Chinese-speaking LEP participants, 14 Spanish-speaking LEP participants, and 17 EMS personnel. Each session lasted about 1.5-2.5 hours, depending on the number of people in the group. Table 2 summarizes the characteristics of participants. The EMS personnel in the study had a mean age of 44.2 years and an average of 17.8 years of experience.

The Chinese-speaking LEP participants had a mean age of 46 years and had lived in the United States for an average of 7.3 years. The Spanish-speaking LEP participants had a mean age of 44.7 years and lived in the United States for an average of 13.9 years. Over half of the Chinese-speaking participants and two-thirds of the Spanish-speaking participants identified themselves as having intermediate level English, both spoken and written.

### Comparison of QuickSpeak and Google Translate

#### Postsession Questionnaire

Of 17 EMS respondents, 53% (n=9) indicated that they preferred QuickSpeak over Google Translate. Some EMS personnel (3/17, 18%) stated that they would like a tool that combines features of both technologies. There was 1 EMS participant who said they would not use either system. In the specific follow-up questions (summarized in Table 3), 76% (13/17) of EMS participants stated that QuickSpeak helped them to get the information needed during the simulation session. Fewer participants (10/17, 59%) reported that Google Translate provided the needed information. All but 1 EMS participant noted that QuickSpeak helped them to communicate with LEP participants. In contrast, only approximately two-thirds of respondents mentioned that Google Translate helped them with communication.

**Table 2:** Participants’ demographics.

Characteristics		Emergency Medical Services (n=17)	Chinese-speaking (n=12)	Spanish-speaking (n=14)
Age (years), mean (SD)		44.2 (9.6)	46.0 (25.1)	44.7 (16.1)
Years of Emergency Medical Services experience, mean (SD)		17.8 (11.9)	N/A^a^	N/A
Years in the United States, mean (SD)		N/A	7.3 (7.5)	13.9 (8.8)
**Education, n (%)**				
	Less than high school or equivalent	0 (0)	0 (0)	5 (36)
	High school graduate or equivalent	2 (12)	5 (42)	4 (29)
	Some college or college graduate	13 (76)	2 (17)	3 (21)
	Graduate or professional degree	2 (12)	3 (25)	2 (14)
	Chose not to answer	0 (0)	2 (17)	0 (0)
**Self-reported English level (spoken), n (%)**				
	Beginner	N/A	4 (33)	3 (21)
	Intermediate	N/A	8 (67)	5 (29)
	Advanced	N/A	0 (0)	3 (21)
	Chose not to answer	N/A	0 (0)	3 (21)
**Self-reported English level (written), n (%)**				
	Beginner	N/A	4 (33)	4 (29)
	Intermediate	N/A	8 (67)	5 (36)
	Advanced	N/A	0 (0)	2 (14)
	Chose not to answer	N/A	0 (0)	3 (21)

^a^N/A: not applicable.

**Table 3:** Emergency Medical Services’ ability to obtain the needed information (n=17).

Follow-up question to Emergency Medical Services		QuickSpeak, n (%)	Google Translate, n (%)
**Able to get the information needed**			
	Yes	13 (76)	10 (59)
	No	2 (12)	5 (29)
	Maybe	0 (0)	1 (6)
	Chose not to answer	2 (12)	1 (6)
**Helped with communication**			
	Yes	16 (94)	10 (59)
	No	0 (0)	3 (18)
	Maybe	1 (6)	3 (18)
	Chose not to answer	0 (0)	1 (6)

**Table 4:** Postsession questionnaire results from limited English proficient participants.

Criteria evaluated		Chinese-speaking (n=12), n (%)				Spanish-speaking (n=14), n (%)		
		QuickSpeak		Google Translate		QuickSpeak	Google Translate	
**Tool useful overall**								
	Yes		12 (100)		5 (42)	11 (79)		2 (14)
	No		0 (0)		6 (50)	1 (7)		8 (57)
	Maybe		0 (0)		1 (8)	2 (14)		4 (29)
**Help to understand Emergency Medical Services**								
	Yes		11 (92)		3 (25)	12 (86)		4 (29)
	No		1 (8)		7 (58)	2 (14)		5 (36)
	Maybe		0 (0)		2 (17)	0 (0)		5 (36)
**Help to speak to Emergency Medical Services**								
	Yes		9 (75)		3 (25)	11 (85)		6 (43)
	No		0 (0)		6 (50)	1 (8)		8 (57)
	Maybe		3 (25)		3 (25)	1 (8)		0 (0)

Table 4 summarizes findings from the postsession questionnaire administered to LEP participants. Similar to EMS, both Chinese-speaking and Spanish-speaking LEP participants clearly favored QuickSpeak. When asked about overall usefulness of the tools, all 12 Chinese-speaking LEP participants and 11 of the 14 Spanish-speaking LEP participants noted that QuickSpeak was useful. There was 1 participant who commented on the necessity of such a tool.

Yes, it was useful at the time. It is necessary when there is no interpreter.

Chinese-speaking, P3

Relatively few LEP participants deemed Google Translate useful (5/12, 42% Chinese-speaking and 2/14,14% Spanish-speaking). Some participants explained that it took too long for EMS personnel to use Google Translate, and they did not feel confident in the quality of the translation.

[Google Translate is] not useful nor pleasant…I could not communicate what I have or what I need. It takes too long for them to ask questions and it does not feel safe or that one is being understood.

Spanish-speaking, P2

Again, similar to responses from EMS participants, the majority of Chinese-speaking and Spanish-speaking LEP participants (11/12, 92% and 12/14, 86%, respectively) thought QuickSpeak helped them understand the EMS personnel’s questions. On the other hand, Google Translate was considered helpful by only 25% (3/12) Chinese-speaking and 29% (4/14) Spanish-speaking LEP participants.

When describing their experience with Google Translate, many LEP participants mentioned difficulty in understanding what was being said due to the poor translation quality, ambiguous meanings, and inappropriate wording.

The more basic ones [questions] yes, but the rest, no. The language, the words or the grammar is not appropriate. The words were not translated correctly.

Spanish-speaking, P4

**Summary of limited English proficient participants’ feedback on problems encountered during simulation sessions.**

QuickSpeak

- restriction on response format (yes or no)
- sound unclear
- slow communication process
- poor quality translation
- difficult to communicate temporal or body position information

Google Translate

- restriction on response format (yes or no)
- unclear questions
- slow communication process
- awkward interaction
- unsafe
- cannot communicate back
- guessing necessary to understand
- poor quality
- difficult to understand
- difficult to ask questions
- culturally inappropriate words
- inconsistent

In addition, some participants mentioned that they had to do a lot of guesswork to make connections between poorly translated words.

Basically I can understand these questions, but with my guesses and understandings.

Chinese-speaking, P6

The use of inappropriate words was also mentioned in relation to QuickSpeak. Spanish-speaking LEP participants, in particular, mentioned the ambiguity of word choices such as “drinking” (beverages or alcohol) or “drug.”

Yes, but I believe that when it asks, “Were you drinking?” it is too general. The word “drug” can be used in a different manner by different people. It could mean: drug (illegal), medication, medicine, or remedy.

Spanish-speaking, P2

The LEP participants also commented on whether the tools helped them to respond or speak to EMS personnel. There were 9 of 12 Chinese-speaking participants and 11 of 14 Spanish-speaking participants who mentioned that QuickSpeak helped them speak to EMS personnel. However, only 3 of 12 Chinese-speaking and 6 of 14 Spanish-speaking participants thought that Google Translate helped them. Many participants experienced difficulty communicating detailed responses with both technologies.

When we answered with more than a yes or no, they looked at us with a face of “What?” showing a question mark face.

Chinese-speaking, P8

During sessions, the research staff noted that when EMS were using the Google Translate tool, they started by typing in open-ended questions. However, because they could not understand the responses given, they evolved to asking more “yes” or “no” questions, similar to QuickSpeak. When typing questions into Google Translate, EMS participants also frequently forgot to add question marks, which affected the interpretation of the translation. Textbox 1 provides a summary of LEP participants’ feedback on the problems encountered during their simulation experience.

As primary users of the tools, the EMS participants were asked to provide feedback on usability and recommendations for improving each of the tools. For Google Translate, some EMS personnel mentioned that having a list of predefined questions (as with QuickSpeak) would be helpful. Specifically, they suggested that 4-5 essential questions be placed on the home screen. Some EMS personnel recommended increasing the size of the speaker button, which when clicked plays translated audio, for better usability. There was 1 EMS participant who mentioned that a better translation accent would aid comprehension. Another recommended that Google Translate create a medical domain-specific translation service.

For the QuickSpeak tool, many EMS personnel suggested adding the ability to type and verbalize their own questions, similar to the free-text ability of Google Translate. Some recommended that the list of predetermined questions follow a more logical flow of normal questioning. Some specific suggestions were: use a decision tree to assist with selecting appropriate questions, show a full-body image on the screen allowing EMS personnel to click body parts and view relevant questions, remove questions from the list once they have been asked, allow EMS personnel to add or modify existing questions loaded in QuickSpeak, and expand the number of languages translated.

Some recommendations applied to both technologies. EMS personnel recommended that both services support voice-operated, two-way translation or communication. They also suggested that actual interactions with LEP patients be audio-recorded for record keeping, education, and accountability. A summary of EMS feedback is provided in Textbox 2.

#### System Usability Scale Score Evaluation

Table 5 shows the results of the SUS for QuickSpeak and Google Translate. The SUS was only administered to EMS participants, as they were the primary user group operating the translation tools. The mean SUS score for QuickSpeak was higher than the score for Google Translate. The difference between the two translation tools was statistically significant (Mann-Whitney U test, z=−2.1; *P*=.04). The results were similar for the Chinese and Spanish simulation sessions. However, EMS personnel who participated in the Chinese sessions rated Google Translate higher than EMS personnel who participated in the Spanish sessions.

**Emergency Medical Services personnel feedback on problems encountered during simulation sessions.**

QuickSpeak

- restriction on response format (yes or no)
- cannot create own questions
- need more questions
- difficult to find a question
- not able to type own questions
- poor organization of question flow
- questions not specific
- low sound volume
- difficult to solicit temporal information
- too many questions

Google Translate

- difficulty understanding limited English proficient responses unless questions posed in yes or no format
- poor quality translation
- slow communication process
- difficult to compose questions
- too much attention directed to the screen

**Table 5:** System Usability Scale scores.

Language group	Tool	System Usability Scale Score^a^		
		Mean (SD)	Median	*P* value
Overall	QuickSpeak	65.3 (13.7)	65.0	.04
	Google Translate	48.4 (25.6)	50.0	
Chinese-speaking participants	QuickSpeak	63.6 (9.8)	65.0	.8
	Google Translate	56.1 (28.1)	62.5	
Spanish-speaking participants	QuickSpeak	66.5 (16.3)	70.0	.02
	Google Translate	43.0 (23.7)	42.5	

^a^Maximum possible score is 100.

## Discussion

### Principal Findings

Our scenario-based evaluation of the two translation tools confirmed a need for better tools to assist in communication between LEP individuals and EMS personnel during medical emergencies. This is consistent with prior study findings showing that EMS personnel often experience frustration with telephone language interpreters and resort to talking with bystanders or using body language and keywords they happen to know [39]. Studies show that LEP individuals who have ad hoc interpreters (bystanders or family members) experience more dissatisfaction than those with medically trained interpreters and are more likely to experience errors that could impact clinical care [40]. Improving translation options is key to overcoming communication barriers and increasing the quality of emergency care for LEP individuals.

Although neither translation tool was considered ideal for use in the field, in our scenarios, LEP and EMS participants clearly preferred the fixed question translations of QuickSpeak over the free-text translation of Google Translate. Both LEP and EMS participants thought the ideal translation tool would have the accuracy and clarity of prefixed questions but the flexibility and potential bidirectional communication of the free-text tools. Google Translate currently allows for bidirectional communication, but inaccuracies in translation were often compounded with the existing system. Communication between EMS and LEP individuals was marginally improved with Google Translate and QuickSpeak; however, inaccuracies and potential for miscommunication are great, and neither tool was considered ready for use in the field, where the risks of any miscommunication or delay are high. Our prior research investigating the potential use of freely available Web-based translation systems, such as Google Translate and Microsoft Translator, indicates that in the area of health, these tools require careful postediting by professional translators to improve accuracy [18,41]. Obviously, this kind of post-editing would not be possible in the field. However, machine translation technology is constantly improving. Google has recently updated their translation system to utilize sophisticated artificial intelligence to produce more accurate language translations [42]. Further evaluation of the use of these tools in health settings is needed as automated language translation technology evolves.

In addition to improving translation accuracy, future translation technologies for emergency response should give particular attention to the needs and design recommendations of LEP individuals and EMS personnel. The EMS personnel voiced concern about operating a device and using the translation technology in the fast-paced, real-life emergency setting. Hands-free, accurate speech recognition technology that can facilitate bidirectional communication would be ideal. EMS participants also expressed a desire for translation answers to be recorded for use in documenting encounters. Archiving of answers associated with personal health information, however, would require careful consideration of Health Insurance Portability and Accountability Act of 1996 policies and rules.

Currently, bilingual staff, interpreters, and language lines that can provide real-time, accurate translations are the gold standard for translation and interpretation in clinical settings. Prior studies have shown that LEP patients experience more satisfaction with telephone interpreters than with family, ad hoc, or no interpreters [40]. However, in informal interviews with EMS personnel, there is a hesitancy to use language lines because of concerns that they take too long and that phone-based interpreters may not accurately assess the situation at hand. Despite these perceived barriers, in the absence of in-person interpreters, language lines continue to be the best method of ensuring fast and accurate translations. As automated language translation technologies continue to evolve, further evaluations will be needed to assess whether they can provide safe, effective communication between English and non-English speakers in the field of emergency response.

### Limitations

The simulation sessions were limited in number, and all took place in King County, Washington, which may limit the generalizability of our results. We evaluated Spanish and Mandarin Chinese, the two most common non-English languages spoken in our region. In general, Google Translate performs better with Spanish than with Chinese or lesser used languages [18,41,43], so it is likely that results may have been different if we had tested different languages. In addition, our study took place at one point in time, and translation tools using statistical machine translation are constantly evolving. Our evaluation of the translation tools was based on simulation sessions between EMS and LEP participants. Although we used scenarios based on actual EMS responses, these sessions took place in a controlled environment and do not accurately reflect the performance of these tools in the field.

### New Contributions to the Literature

In the context of a growing LEP population in the United States and disparities in medical care resulting from language barriers, improving translation technologies is critical. In a recent review, Tate (2015) concludes that there are “substantial gaps in understanding the interaction between language barriers and prehospital care” [44]. Our study sheds light on the challenges of the use of new translation technologies in the prehospital, emergency care setting. While there is a significant need for translation tools to assist in translations in these settings, we need to continue to evaluate automated translation technologies as they evolve, to determine how they compare to more traditional phone interpreter services in terms of acceptability, accuracy, and efficiency.

## Appendix

Multimedia Appendix 1 Scenario pairs used for the simulation sessions.

Multimedia Appendix 2 Post-Session Questionnaire.

## Abbreviations

EMS: Emergency Medical Services
LEP: limited English proficient
SMT: statistical machine translation
SUS: System Usability Scale
