# Supplementary material for: Evaluating the Usefulness of Translation Technologies for Emergency Response Communication: A Scenario-Based Study
Source: JMIR Public Health Surveill. 2019 Jan 28;5(1):e11171. doi: 10.2196/11171 (PMC6369422; doi:10.2196/11171)
Supplement: Multimedia Appendix 1 [file publichealth_v5i1e11171_app1.pdf]

Appendix I. Scenario pairs used for the simulation sessions.

**Scenario 1-A:**<br><br>You are a 45-year-old man who has bad stomach pain. You are laying down holding your stomach because it is difficult to walk. You have:<br><br>· Severe abdominal pain, cold sweating, and nausea just after breakfast this morning<br>· Trouble walking<br>· Had an upset stomach every morning for the past 3 weeks<br>· Dark brown stool for the past 3 days<br>· Been smoking for 10 years<br><br>Medications: Zantac twice a day for 1 week, irregularly<br><br>Allergies: None<br><br>Support person role: The man's wife, who can help answer questions if needed.
**Scenario 1-B:**<br><br>You are a 35-year-old woman who has abdominal pain that has gotten worse the past 3 days. You are laying down and holding your right side because the pain in your stomach makes it difficult to walk. You have:<br><br>· Pain that moved from the umbilical area to RLQ area and got worse this morning<br>· Difficulty walking due to pain<br>· Mild fever<br>· Vomiting<br>· A history of childhood surgery for a hernia<br><br>Medications: Multivitamin with iron once daily<br><br>Allergies: NSAIDs, like ibuprofen<br><br>Support person role: The woman's husband, who can help answer questions if needed.

Scenario 2-A:

You are a 30-year-old woman who’s right side of your back has hurt worse and worse the past 3 days. You are sitting down holding the right side of your lower back. You have:

- Burning feeling while urinating for the past week
- Pain and difficulty urinating
- Fever
- Chills and shaking
- Vomiting
- A history of diabetes

Medications: Insulin

Allergies: None

Support person role: The woman's roommate, who can help answer questions if needed.

Scenario 2-B:

You are a 30-year-old man with sudden back pain for the past hour. You are sitting down holding your lower back with both hands. You have:

- Vomited several times and now just nausea
- Weakness and pale skin
- Restless appearance
- Right flank pain

Medications: herbal medication for back pain

Allergies: NSAIDs, like ibuprofen

Support person role: The man's roommate, who can help answer questions if needed.

**Scenario 3-A:**<br><br>You are a 45-year-old man with chest pain this week. You are sitting down holding your chest in pain. You have:<br>· Tightness in your chest<br>· Cold sweating<br>· Nausea<br>· A history of smoking<br><br>Medications: Nil<br><br>Allergies: Aspirin<br><br>Bystander role: The man's wife, who can help answer questions if needed.
**Scenario 3-B:**<br><br>You are a 30-year-old man with chest pain for the past 3 days that started suddenly when you coughed. You are sitting down holding the left side of your chest in pain. You have:<br><br>· Vague pain on your left side that gets worth when you take a deep breath<br>· Shortness of breath<br>· A history of playing sports<br>· Prior surgery for left collapsed lung<br><br>Medications: None<br><br>Allergies: None<br><br>Bystander role: A friend who can help answer questions if needed.
