# Supplementary material for: Evaluating the Usefulness of Translation Technologies for Emergency Response Communication: A Scenario-Based Study
Source: JMIR Public Health Surveill. 2019 Jan 28;5(1):e11171. doi: 10.2196/11171 (PMC6369422; doi:10.2196/11171)
Supplement: Multimedia Appendix 2 [file publichealth_v5i1e11171_app2.pdf]

Appendix 2. Post-Session Questionnaire.

**EMS Personnel:**
• If you have used both translation tools today, which did you prefer and why?<br>• Were you able to get the information you needed? Why or why not?<br>• Did you think the translation tool helped with communication? Why or why not?<br>• What problems did you encounter?<br>• What did the translation tool do well?<br>• If you were trying to make this translation tool better, what changes would you make?<br>• Have you ever needed translation or interpretation during a medical emergency? If so, please describe your experience.
**LEP Participants:**
• How would you describe your experience trying to use this translation tool? Was it useful?<br>• Did you understand the EMT's questions? If not, please explain why.<br>• Was the EMT able to understand you with the help of the translation tool? If not, please explain why.<br>• Have you ever needed translation or interpretation during a medical emergency? If so, please describe your experience.
